# Physical activity patterns from mid- to late adulthood and risk of sarcopenia in older adults: the HUNT study

**DOI:** 10.1186/s12877-026-07747-6

**Published:** 2026-06-04

**Authors:** Karina Hammer Tømmerdal, Javaid Nauman, Erik Madssen, Jari Antero Laukkanen, Arnt Erik Tjønna, Ulrik Wisløff, Jonathan Berg

**Affiliations:** 1 Cardiac Exercise Research Group, Department of Circulation and Medical Imaging, Faculty of Medicine and Health Sciences, Norwegian University of Science and Technology, Trondheim, Norway; 2 Clinic of Cardiology and Thoracic Surgery, St. Olavs Hospital, Trondheim, Norway; 3 Institute of Public Health, College of Medicine and Health Sciences, United Arab Emirates University, Al-Ain, United Arab Emirates; 4 Institute of Clinical Medicine, Department of Medicine, University of Eastern Finland, Kuopio, Finland; 5 Department of Medicine, Wellbeing Services County of Central Finland, Jyväskylä, Finland; 6 Centre for Research on Exercise, Physical Activity and Health, School of Human Movement and Nutrition Sciences, The University of Queensland, Brisbane, QLD Australia

**Keywords:** Physical Activity, Sarcopenia, Longitudinal, Aging, Muscle Strength, Muscle Mass

## Abstract

**Background:**

While cross-sectional studies suggest that physical activity (PA) is associated with a lower risk of sarcopenia, the long-term PA patterns remain poorly characterized. We aimed to examine whether PA patterns across mid- to late adulthood are associated with sarcopenia in older age.

**Methods:**

We included 4702 individuals (mean age 76.4 ± 5.0 years, 53% female) from the HUNT4 (Trøndelag Health Study) 70+ cohort with PA data across three prior waves (HUNT1–3). PA was categorized as active or inactive at each time point (1984-86, 1995-97, 2006-08) according to the World Health Organization 2020 guidelines. Sarcopenia at HUNT4 (2017-19) was defined according to the European Working Group on Sarcopenia (EWGSOP2) criteria as probable (low strength) or confirmed (low strength and low muscle mass). Logistic regression analyses were used to examine associations between long-term PA patterns and sarcopenia.

**Results:**

PA showed a clear inverse dose-response relationship with sarcopenia, with stronger associations for confirmed than probable sarcopenia. Compared with persistent inactivity, being active at all three time points was associated with 78% lower odds of confirmed sarcopenia at HUNT4 (OR 0.22, 95% CI: 0.08-0.66). Participants active at two and one time points had 40% (OR 0.60, 0.38-0.95) and 26% (OR 0.74, 0.55-1.00) lower odds, respectively. Associations varied by PA pattern, with lower odds observed among those becoming active (OR 0.57, 0.39–0.84) but not among those becoming inactive.

**Conclusion:**

Long-term PA shows a clear dose–response relationship with sarcopenia risk in later life. While sustained activity across adulthood confers the greatest benefit, initiating PA even later in life remains associated with lower odds of sarcopenia.

**Supplementary Information:**

The online version contains supplementary material available at 10.1186/s12877-026-07747-6.

## Background

Sarcopenia is a progressive, age-related skeletal muscle disorder characterized by loss of muscle strength, function, and/or mass [1–4]. It is associated with adverse outcomes such as falls, fractures, morbidity, and mortality [2]. The prevalence of sarcopenia rises steeply with age, affecting approximately 40–60% of individuals aged 85 years and older, and is projected to increase substantially with population aging [5, 6]. Physical activity (PA) is a modifiable risk factor for sarcopenia prevention. The World Health Organization (WHO) recommends that adults engage in at least 150 min of moderate-intensity PA or 75 min of vigorous-intensity PA per week, or an equivalent combination, in addition to muscle-strengthening activities on two or more days per week [7]. However, approximately one-third of the global adult population did not meet these recommendations in 2022, and the prevalence of physical inactivity increases markedly with age [8].

Cross-sectional studies and meta-analyses consistently demonstrate that higher levels of PA are associated with lower risk of sarcopenia and more favorable muscle health outcomes [9–11]. Randomized controlled trials have further established that resistance training improves muscle strength and physical function in older adults [12–15], and resistance training is consequently recommended as the primary exercise modality in international sarcopenia management guidelines [16]. However, these findings are largely derived from structured resistance training interventions, whereas aerobic activities constitute the dominant mode of PA in free-living populations. Indeed, approximately 75–85% of adults globally do not meet muscle-strengthening recommendations, with adherence declining further with age [17–19], reflecting limited resistance training uptake in real-world settings.

PA is a dynamic behavior that changes across adulthood in response to aging, life events, and health transitions [20]. However, longitudinal studies on PA and sarcopenia are scarce and have typically relied on single baseline assessments, short follow-up periods, or individual outcomes such as muscle mass or strength rather than sarcopenia defined using contemporary criteria [21–26]. Consequently, it remains unclear whether long-term patterns, temporal changes, or cumulative exposure to PA are most relevant for sarcopenia risk in later life.

Therefore, the primary aim of this study was to examine the association between long-term PA patterns from mid-to late adulthood and sarcopenia in later life. As a secondary aim, we examined the association between time-updated PA and subsequent sarcopenia using repeated measures analyses. We hypothesized that sustained adherence to PA recommendations across adulthood would be associated with lower sarcopenia risk in later life.

## Methods

### Study population and design

**Fig. 1:**
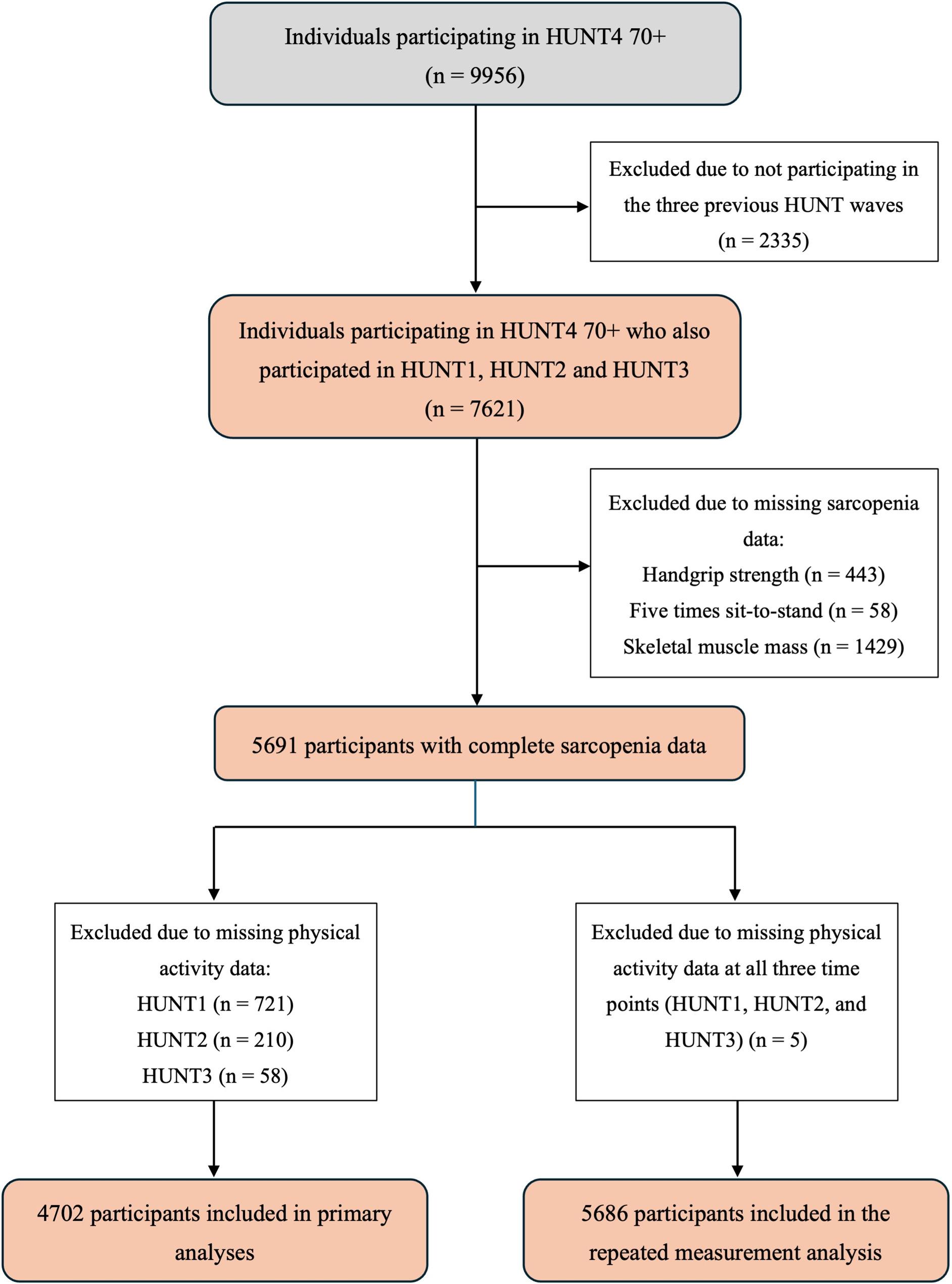
Flow chart illustrating the flow of participants in the study

This longitudinal cohort study utilized data from the Trøndelag Health Study (HUNT), a Norwegian population-based study in which the entire population of Nord-Trøndelag County aged 20 years or older was invited to participate. To this date, it has comprised four waves where participants are followed longitudinally between surveys. The details of the HUNT study have been explained elsewhere [27]. In brief, HUNT1 was undertaken in 1984–1986 with 77,202 participants (89.4% of those invited), HUNT2 in 1995–1997 with 65,228 participants (69.5%), HUNT3 in 2006–2008 with 50,800 participants (54.1%), and HUNT4 (2017–2019) with 56,042 participants (54.0%) [27]. The fourth wave also comprised a separate survey for participants aged ≥ 70 years (HUNT4 70+) and included 9956 participants from Nord-Trøndelag County [27]. The present study included data from participants in HUNT4 70 + who also participated in the three previous HUNT waves (*n* = 7621 participants). We excluded participants with missing data on handgrip strength (HGS), the five times sit-to-stand (5-STS) test, or skeletal muscle mass, yielding 5691 participants. For the primary analysis, we further excluded participants without complete PA data from HUNT1, HUNT2, and HUNT3, resulting in a total of 4702 participants (Fig. 1). For the repeated measurement analysis, we included participants with PA data from at least one time point (*n* = 5686). All participants provided informed written consents, and the study was approved by the Regional Committees for Medical and Health Research Ethics (REC Midt, 2021/401190).

### Physical activity

PA data was obtained from self-reported questionnaires in the HUNT1, HUNT2, and HUNT3 surveys capturing overall PA.

In HUNT1 and HUNT3, participants reported exercise frequency, intensity, and duration. Frequency (“How often do you exercise?”) was converted from categorical responses and averaged to days/week (Never = 0; Less than once per week = 0.5; Once per week = 1; 2–3 times a week = 2.5; Nearly every day = 5). Intensity (“How hard do you exercise?”) was categorized as light (“No sweat or heavy breathing”), moderate (“Sweating and heavy breathing”), or hard (“Pushing myself to exhaustion”). Duration (“How long do you exercise each time?”) was converted from categorical responses to average minutes (< 15 min = 7.5; 16–30 min = 22.5; 30–60 min = 45; > 1 h = 60).

In the HUNT2 survey, participants reported weekly time spent performing light and hard-intensity exercise, with options “0”, “Less than 1 hour”, “1–2 hours”, or “3 hours or more”. These categorical responses were converted to average weekly minutes (0 = 0; Less than 1 h = 30 min; 1–2 h = 90 min; 3 h or more = 180 min).

Weekly minutes of moderate-to-vigorous PA were then calculated for each participant at all three HUNT surveys by multiplying the average frequency and average duration for HUNT1 and HUNT3, and by using the reported average weekly minutes of hard-intensity exercise for HUNT2. Participants were subsequently classified according to WHO 2020 guidelines [7] into two groups: “Below recommendations” (< 150 min of moderate or < 75 min of vigorous intensity PA/week) and “At or above recommendations” (≥ 150 min of moderate or ≥ 75 min of vigorous intensity PA/week).

For the primary analysis, participants were categorized into one of five mutually exclusive PA pattern groups based on their data from HUNT1, HUNT2 and HUNT3: always inactive (inactive at all three time points), becoming inactive (active at HUNT1 but inactive by HUNT3), fluctuating (a non-sequential pattern: active-inactive-active or inactive-active-inactive), becoming active (inactive at HUNT1 but active by HUNT3) or always active (active at all three time points).

### Sarcopenia

Information regarding sarcopenia was obtained from clinical examinations conducted by trained personnel at HUNT4 70+. We followed the conceptual definition of the Delphi Consensus from the Global Leadership Initiative in Sarcopenia [28], emphasizing reduction in muscle strength and muscle mass as key components of sarcopenia. We categorized all participants as having no sarcopenia, probable sarcopenia (low muscle strength), or confirmed sarcopenia (low muscle strength and low muscle mass) according to the updated European Working Group on Sarcopenia (EWGSOP2) guidelines [2]. Muscle strength was estimated using both a HGS test to assess upper-body strength and the 5-STS test to assess lower-body strength. For maximal HGS, participants conducted three attempts with each hand using a Jamar hand-held dynamometer, and the best attempt was recorded in kg to the nearest decimal. For 5-STS, participants were instructed to fold their arms across their chest and then stand up and sit down as quickly as possible, repeating this five times. The buttocks had to touch the chair each time the participant sat down for the attempt to be approved, and the legs were not to press against the chair. We recorded the total seconds spent on the test to the nearest decimal. Skeletal muscle index was calculated as skeletal muscle mass (estimated by InBody770, Biospace, Seoul, South Korea) divided by body height and reported to the nearest two decimals in kg/m.

The EWGSOP2 guidelines recommend deriving normative values from a young, healthy reference population, with cut-off values set at 2-2.5 standard deviations (SD) below the mean reference value [2]. Following these recommendations, we used sex-specific reference values representing peak adult strength from a healthy Norwegian population [29, 30]. We defined cut-off points at 2 SD below the mean, corresponding to HGS values of < 22.2 kg for females and < 37.7 kg for males, and 5-STS values of > 14.4 and > 13.3 s, respectively. The corresponding cut-offs for muscle mass were < 7.66 kg/m^2^ for females and < 9.54 kg/m^2^ for males. Participants unable to complete the 5-STS test were conservatively classified as having low muscle strength, based on the assumption that inability to perform the test reflects impaired lower-body function. These individuals were therefore included in the probable sarcopenia category rather than excluded, to reduce potential bias associated with excluding participants with poor physical function. Participants unable to complete the HGS test were excluded, as the reason for non-completion was not recorded, and missing data could reflect factors unrelated to muscle strength, such as hand injury or pain, rather than true weakness.

### Other variables

Height and weight were measured to the nearest decimal using SECA 217 and InBody770, respectively. Body mass index (BMI) was calculated as kg/m^2^. All anthropometric measurements were performed with participants in an upright standing position, wearing light indoor clothing and no shoes. Blood pressure was measured three times at 1-minute intervals using an automated recorder with participants in a seated position. The mean of the second and third readings was used for analysis. Hypertension was defined as a systolic blood pressure of ≥ 140 mmHg, a diastolic blood pressure ≥ 90, and/or self-reported use of blood pressure medications. We obtained information regarding covariates from questionnaires, including age (years), sex (male/female), smoking status (current/previous daily smoker versus never), weekly alcohol consumption (categorized as < 2, 2–3, ≥ 4 weekly units), self-reported diabetes (yes/no) and highest completed education (7–10 years, 11–13 years, > 13 years). Self-reported health status was dichotomized as “healthy” (no history of angina pectoris, myocardial infarction, heart failure, atrial fibrillation, stroke, and/or chronic obstructive pulmonary disease (COPD)) or “unhealthy”. Diet quality was defined according to the contemporary recommendations for each HUNT wave. For HUNT 2, diet was categorized as “poor” if participants regularly used dairy butter and/or hard margarine as cooking fat, and “good” otherwise. For HUNT3, diet was categorized by weekly intake of fruit, berries, and vegetables, with “poor” defined as less than 7 times per week and “good” as at least 7 times per week.

### Statistical analyses

Descriptive data are presented as mean (SD) for continuous variables and as number (%) for categorical variables. The association between PA patterns and the odds of having sarcopenia was examined using binary logistic regression. The “always inactive” group was used as the reference category. Basic models (model 1) were adjusted for sex and age, while model 2 was further adjusted for self-reported health status, education level, smoking status, alcohol consumption, diabetes, hypertension, BMI, and diet quality. Covariates were selected a priori based on established associations with both PA and sarcopenia in the literature [2, 31]. Formal tests for interaction between PA patterns and sex, using multiplicative interaction terms, were conducted in all primary models. No statistically significant interactions were identified (all p for interaction > 0.05); therefore, sex was included as a covariate in all models rather than used as a stratification variable. For descriptive purposes, BMI is presented as four categories (underweight, normal weight, overweight, and obese) in Table 1 and Supplementary Material 1. In regression analyses, the underweight and normal weight groups were combined into a single reference category (normal weight) due to the small number of participants in the underweight group.

**Table 1:** Participant characteristics at HUNT4 stratified by sarcopenia group and sex

Variable	Men (*n* = 2234)			Women (*n* = 2468)		
	No sarcopenia (*n* = 1308)	Probable sarcopenia (*n* = 704)	Confirmed sarcopenia (*n* = 222)	No sarcopenia (*n* = 1613)	Probable sarcopenia (*n* = 719)	Confirmed sarcopenia (*n* = 136)
Age (years)	75.9 (4.6)	78.5 (5.9)	83.2 (5.9)	75.7 (4.5)	78.9 (5.7)	81.4 (6.6)
Years of completed education, n (%)						
7–10 years 11–13 years > 13 years * Missing*,* n (%)*	341 (18.2) 912 (48.7) 621 (33.1) *10 (0.5)*	73 (28.6) 139 (54.5) 43 (16.9) *2 (0.8)*	28 (30.4) 51 (55.4) 13 (14.1) *1 (1.1)*	583 (29.4) 890 (44.9) 507 (25.6) *16 (0.8)*	177 (44.6) 164 (41.3) 56 (14.1) *5 (1.2)*	34 (50.0) 26 (38.2) 8 (14.1) *2 (2.9)*
Current or previous daily smoker, n (%)	742 (57.1)	446 (64.3)	138 (62.2)	809 (50.7)	334 (47.2)	71 (53.0)
* Missing*,* n (%)*	*8 (0.6)*	*10 (1.4)*	*0 (0.0)*	*16 (1.0)*	*11 (1.5)*	*2 (1.5)*
Weekly alcohol consumption, n (%)						
Low: < 2 units	403 (33.1)	250 (40.3)	99 (54.1)	719 (53.3)	363 (64.6)	66 (61.1)
Moderate: 2–3 units	417 (34.3)	209 (33.7)	49 (26.8)	402 (29.8)	131 (23.3)	32 (29.6)
High: ≥ 4 units	396 (32.6)	162 (26.1)	35 (19.1)	228 (16.9)	68 (12.1)	10 (9.3)
* Missing*,* n (%)*	*92 (7.0)*	*83 (11.8)*	*39 (17.6)*	*264 (16.4)*	*157 (21.8)*	*28 (20.6)*
Hypertension, n (%)	938 (82.0)	519 (83.3)	150 (80.2)	1163 (82.4)	540 (82.6)	104 (83.9)
* Missing*,* n (%)*	*164 (12.5)*	*81 (11.5)*	*35 (15.8)*	*202 (12.5)*	*65 (9.0)*	*12 (8.8)*
Diabetes (HUNT3), n (%)	69 (5.3)	76 (10.8)	17 (7.7)	71 (4.4)	54 (7.5)	6 (4.4)
Body mass index (kg/m^2^)	27.2 (3.3)	28.4 (3.7)	24.2 (2.5)	26.7 (4.2)	28.3 (4.6)	22.3 (3.3)
Body mass index (kg/m^2^) category, n (%)						
Underweight: < 18.5	1 (0.1)	0 (0.0)	2 (0.9)	17 (1.1)	0 (0.0)	18 (13.2)
Normal weight: 18.5–24.9	347 (26.5)	115 (16.3)	139 (62.6)	577 (35.8)	168 (23.4)	91 (66.9)
Overweight: 25-29.9	716 (54.7)	388 (55.1)	79 (35.6)	671 (41.6)	333 (46.3)	26 (19.1)
Obese: ≥ 30	244 (18.7)	201 (28.6)	2 (0.9)	348 (21.6)	218 (30.3)	1 (0.7)
Handgrip strength (kg)	46.7 (6.2)	38.0 (7.4)	33.2 (5.9)	28.3 (4.0)	22.5 (5.0)	19.8 (4.2)
Five times sit-to-stand (seconds)	9.6 (1.9)	13.4 (3.9)	13.2 (4.4)	10.5 (2.0)	14.6 (4.4)	13.7 (3.8)
* Not able to perform test*,* n (%)*	0 (0.0)	62 (8.8)	29 (13.1)	0 (0.0)	114 (15.9)	15 (11.0)
Skeletal muscle index (kg/m^2^)	10.83 (0.87)	10.71 (0.80)	9.02 (0.45)	8.91 (0.82)	8.85 (0.79)	7.25 (0.37)
Gait speed (m/s)	1.09 (0.24)	0.94 (0.22)	0.90 (0.23)	1.07 (0.23)	0.88 (0.24)	0.87 (0.26)
* Not able to perform test*,* n (%)*	0 (0.0)	8 (1.1)	2 (0.9)	1 (0.1)	14 (1.9)	4 (2.9)
* Missing*,* n (%)*	*34 (2.6)*	*9 (4.1)*	*5 (2.3)*	*45 (2.8)*	*18 (2.5)*	*1 (0.7)*

Data are presented as mean (SD) unless otherwise stated

In a separate analysis, we used logistic regression with repeated observations to assess the association between PA at each specific study wave and subsequent sarcopenia. The dataset was restructured into a long format with one observation per participant per study wave (HUNT1, HUNT2, HUNT3). The outcome variable was the presence of sarcopenia at the subsequent HUNT4 survey. The primary exposure was the time-updated PA level (categorized) reported at each prior wave (HUNT1-HUNT3). All time-varying confounders (e.g., BMI, smoking status) were similarly updated to their values at the corresponding wave. We accounted for the non-independence of multiple observations from the same participant by using cluster-robust standard errors. This approach estimates the odds of sarcopenia at HUNT4 associated with PA level at any given prior assessment, utilizing all available data while properly accounting for within-person correlation.

To assess the robustness of our findings, we performed several sensitivity analyses. First, to address potential reverse causation whereby pre-existing conditions could influence PA level, we repeated the primary analysis, who self-reported no history of cardiovascular disease (CVD) or COPD. Second, to further minimize bias from underlying mobility impairment, we excluded individuals reporting motor ability impairment at HUNT3. Third, to evaluate potential measurement error due to differences in PA questions between HUNT2 and HUNT1 and HUNT3, we repeated the primary analysis using PA data exclusively from HUNT1 and HUNT3. Fourth, we performed a sensitivity analysis in which participants who were unable to complete the HGS test were classified as having low muscle strength (probable sarcopenia) rather than excluded. Finally, to account for potential bias due to missing data, we performed multiple imputation using the Fully Conditional Specification (FCS) method to generate 10 imputed datasets on all potential confounders. The following variables from HUNT3 contained missing values: BMI (11, 0.2% missing), hypertension (16, 0.3% missing), smoking status (47, 1.0% missing), alcohol consumption category (238, 5.1% missing), and education level (36, 0.8% missing). The imputation model incorporated all covariates included in the primary analysis. We applied logistic regression for binary variables and linear regression for continuous variables. The primary analysis was subsequently repeated on each imputed dataset.

Statistical analyses were performed using IBM SPSS Statistics 29.0.2.0 [20] (Armonk, New York, USA: IBM Corp), and Stata for Windows (version 16, StataCorp LLC, TX, USA). A two-sided p-value of ≤ 0.05 was considered statistically significant.

## Results

### Subject characteristics

Table 1 presents the baseline characteristics of the 4702 included participants. The mean age of the participants was 76.4 years (± 5.0), and 52.5% were females. See Additional file 1 in Supplementary Material for characteristics at HUNT4 for all those who participated in HUNT4 70 + and the first three HUNT waves (*n* = 7621), and Additional file 2 in Supplementary Material for a comparison of included versus excluded (*n* = 2919) participants.

### Association of physical activity patterns with the odds of sarcopenia

Compared with individuals who remained inactive across all three time points, both persistently active individuals and those who became active had lower odds of confirmed sarcopenia (Table 2). In the fully adjusted model, persistently active individuals had a 78% reduction in the odds of confirmed sarcopenia (OR 0.22, 95% CI 0.08–0.66), while those who became active experienced a 43% reduction (OR 0.57, 0.39–0.84).

**Table 2:** Physical activity patterns and the odds of sarcopenia

PHYSICAL ACTIVITY PATTERNS HUNT1, HUNT2, HUNT3	ODDS OF SARCOPENIA AT HUNT4			
	Probable sarcopenia normative values		Confirmed sarcopenia normative values	
	OR (95% CI) Model 1	OR (95% CI) Model 2	OR (95% CI) Model 1	OR (95% CI) Model 2
Always inactive (*n* = 2687)	1.00 (Reference)	1.00 (Reference)	1.00 (Reference)	1.00 (Reference)
Becoming inactive ^a^ (*n* = 143)	0.74 (0.51–1.08)	0.79 (0.54–1.16)	0.71 (0.35–1.44)	0.70 (0.33–1.47)
Fluctuating ^b^ (*n* = 761)	0.76 (0.64–0.92)	0.84 (0.70–1.01)	0.94 (0.68–1.29)	0.84 (0.60–1.17)
Becoming active ^c^ (*n* = 686)	0.66 (0.55–0.80)	0.75 (0.61–0.91)	0.68 (0.47–0.98)	0.57 (0.39–0.84)
Always active (*n* = 108)	0.63 (0.41–0.98)	0.79 (0.50–1.23)	0.33 (0.12–0.94)	0.22 (0.08–0.66)

^a^ = active at HUNT1 but inactive by HUNT3 (either active or inactive at HUNT2)

^b^ = a non-sequential pattern: active-inactive-active or inactive-active-inactive

^c^ = inactive at HUNT1 but active by HUNT3 (either active or inactive at HUNT2)

Model 1: adjusted for sex (male, female) and age

Model 2: further adjusted for BMI category, education category, self-reported health status (history of angina pectoris, myocardial infarction, heart failure, atrial fibrillation, stroke, or COPD), smoking (yes, no), hypertension (yes, no), diabetes (yes, no), weekly alcohol consumption, and diet (healthy, poor)

*CI *Confidence interval

For probable sarcopenia, becoming active conferred a 25% reduction in odds (OR 0.75, 0.61–0.91) in the fully adjusted model. Persistent PA was associated with 37% lower odds in the age- and sex-adjusted model (OR 0.63, 0.41–0.98), but not in the fully adjusted model. Becoming inactive or having fluctuating PA levels showed no consistent associations with either probable or confirmed sarcopenia.

We observed a clear inverse dose-response relationship between the number of time points where participants met PA recommendations and the odds of having confirmed sarcopenia at HUNT4 (Table 3). Compared to persistently inactive individuals, the odds of confirmed sarcopenia in the fully adjusted model decreased progressively with greater cumulative PA exposure: a 26% reduction for those active at one time point (OR 0.74, 0.55-1.00), 40% reduction for two time points (OR 0.60, 0.38–0.95) and a 78% reduction for all three time points (OR 0.22, 0.08–0.66).

**Table 3:** Association of the number of active time points with the odds of sarcopenia at HUNT4

ACTIVE TIME POINTS HUNT1, HUNT2, HUNT3	ODDS OF SARCOPENIA AT HUNT4			
	Probable sarcopenia normative values		Confirmed sarcopenia normative values	
	OR (95% CI) Model 1	OR (95% CI) Model 2	OR (95% CI) Model 1	OR (95% CI) Model 2
Active = 0 (*n* = 2687)	1.00 (Reference)	1.00 (Reference)	1.00 (Reference)	1.00 (Reference)
Active = 1 (*n* = 1158)	0.77 (0.66–0.90)	0.84 (0.71–0.98)	0.84 (0.63–1.11)	0.74 (0.55–1.00)
Active = 2 (*n* = 432)	0.59 (0.46–0.74)	0.69 (0.54–0.87)	0.71 (0.46–1.09)	0.60 (0.38–0.95)
Always active (*n* = 108)	0.63 (0.40–0.98)	0.78 (0.50–1.23)	0.33 (0.12–0.94)	0.22 (0.08–0.66)

Model 1: adjusted for sex (male, female) and age

Model 2: further adjusted for BMI category, education category, self-reported health status (history of angina pectoris, myocardial infarction, heart failure, atrial fibrillation, stroke, or COPD), smoking (yes, no), hypertension (yes, no), diabetes (yes, no), weekly alcohol consumption, and diet (healthy, poor)

*CI* Confidence interval

### Repeated measurement analysis

To evaluate the overall protective effect of PA regardless of timing, we conducted a repeated measurement analysis comparing persistently inactive participants with those active at one or more time points (Table 4). In the fully adjusted model, being active (meeting PA recommendations) at any time point was associated with a 42% reduction in the odds of confirmed sarcopenia (OR 0.58, 0.35–0.95). For probable sarcopenia, being active at one or more time points showed a significant protective association in the age- and sex-adjusted model (OR 0.71, 0.63–0.79) but not in the fully adjusted model.

**Table 4:** Repeated measurement analysis of the association between physical activity and the odds of sarcopenia

ACTIVE TIME POINTS HUNT1, HUNT2, HUNT3	ODDS OF SARCOPENIA AT HUNT4			
	Probable sarcopenia normative values		Confirmed sarcopenia normative values	
	OR (95% CI) Model 1	OR (95% CI) Model 2	OR (95% CI) Model 1	OR (95% CI) Model 2
Inactive at all time points	1.00 (Reference)	1.00 (Reference)	1.00 (Reference)	1.00 (Reference)
≥ 1 active time point	0.71 (0.63–0.79)	0.95 (0.73–1.24)	0.71 (0.58–0.86)	0.58 (0.35–0.95)

Model 1: adjusted for sex (male, female) and age

Model 2: further adjusted for BMI category, education category, self-reported health status (history of angina pectoris, myocardial infarction, heart failure, atrial fibrillation, stroke, or COPD), smoking (yes, no), hypertension (yes, no), diabetes (yes, no), weekly alcohol consumption, and diet (healthy, poor)

*CI *Confidence interval

### Sensitivity analyses

When excluding individuals with self-reported CVD, COPD, and mobility limitations, the direction of associations between PA and both probable and confirmed sarcopenia remained consistent, although effect estimates were slightly attenuated (Additional files 3 and 4 in Supplementary Material). Excluding PA data from HUNT2 or using 10 multiply imputed datasets had minimal impact on point estimates (Additional files 5 and 6 in Supplementary Material). In a further sensitivity analysis, participants who were unable to complete the HGS test were classified as having low muscle strength rather than excluded. Results from this analysis (Additional file 7 in Supplementary Material) were similar to the primary findings.

## Discussion

In this prospective cohort study of 4702 participants aged ≥ 70 years, both sustained PA and PA initiation in mid-to-late adulthood were associated with lower odds of sarcopenia later in life. Associations were stronger for confirmed than probable sarcopenia. We observed an inverse dose-response relationship, with progressively lower odds of sarcopenia for each additional time point at which participants met PA recommendations. Furthermore, individuals who were initially below recommended PA levels but later met PA recommendations had significantly lower odds of sarcopenia.

This study extends existing literature by examining long-term PA patterns over more than two decades using repeated-measures data. This approach captures sustained PA, changes over time, and cumulative exposure in relation to sarcopenia later in life. With longitudinal PA data from over 4500 participants measured at three distinct time points, our findings provide insight into how dynamic PA behaviors across adulthood relate to sarcopenia risk. The association between PA and sarcopenia observed here aligns with a 2017 systematic review and meta-analysis, which reported that PA was linked to a 55% lower odds of sarcopenia later in life [11]. Furthermore, consistent with our findings of progressive decrease in the likelihood of developing sarcopenia for each time point participants met the PA recommendations, more recent meta-analytic evidence has demonstrated an inverse-dose relationship between PA and sarcopenia [9].

Beyond cumulative exposure, we also observed a time-dependent association. Among participants with a similar number of time points meeting PA recommendations, lower odds of sarcopenia were observed only among those who met recommendations at the most recent assessment. Although recent PA may be particularly important for maintaining muscle mass and strength, this relationship may also reflect reverse causation, whereby early declines in muscle strength potentially lead to reductions in PA before the development of sarcopenia [32]. The importance of maintaining sufficient PA aligns with previous research on sarcopenia and its components, including muscle strength and muscle mass [21–24]. For example, the EpiFloripa Elderly Study reported higher odds of sarcopenia among women who remained inactive or transitioned to inactivity, compared with those who remained active or transitioned to meeting PA recommendations over 5 years [21]. We extend the EpiFloripa findings by demonstrating that this relationship persists over a 20-year follow-up period and by applying contemporary EWGSOP2 criteria [33], which include both muscle strength and muscle mass rather than just muscle mass.

Notably, we observed a stronger link between PA and confirmed versus probable sarcopenia, with odds ratios of greater magnitude and associations that were more robust to covariate adjustment. This discrepancy likely reflects both phenotype specificity and measurement properties inherent to the sarcopenia definitions [2, 34]. While concurrent deficits in muscle strength and mass represent a more specific muscle-wasting phenotype characteristic of confirmed sarcopenia [2], reduced strength alone may arise from transient non-muscular factors, including pathological conditions, acute illness, pain, or neurological impairments [34, 35]. Recent studies suggest a non-linear relationship between muscle mass and strength, with strength declining 2–5 times faster than muscle mass after age 75 [36, 37]. Consequently, the greater heterogeneity in the probable sarcopenia group may increase susceptibility to misclassification and attenuate the observed association with long-term PA [38]. Conversely, the additional requirement of low muscle mass in confirmed sarcopenia may better capture a pathological state more consistently linked to sustained physical inactivity over time [2, 39, 40].

Our analyses were adjusted for several factors associated with both PA and sarcopenia, including age, sex, BMI, smoking, alcohol consumption, diabetes, hypertension, educational attainment, self-reported health status, and diet quality [2, 31]. Adjustment for these variables attenuated several associations, particularly for probable sarcopenia, suggesting that part of the observed relationship between PA and muscle strength reflects differences in overall health status and lifestyle. In contrast, associations with confirmed sarcopenia were less affected by adjustment, indicating that the relationship between sustained PA and muscle mass appeared more robust to confounding.

### Strengths and limitations

The strengths of this study include the long follow-up period and the repeated PA measurements over two decades. The large sample size, balanced sex distribution (52.5% females), and high participation rate enhance the generalizability of the results to community-dwelling adults. Nord-Trøndelag is considered a representative region of the Norwegian population [25], further improving generalizability. All participants aged ≥ 20 years in Nord-Trøndelag were invited, thus minimizing the potential for selection bias. The use of standardized, objective measurements for sarcopenia diagnosis, including dynamometry for HGS and bioelectrical impedance analysis for muscle mass, enhances the validity and reproducibility of our findings. Also, despite slight attenuations in effect estimates, the direction of associations remained across sensitivity analyses, supporting the robustness of our findings.

This study has several limitations. First, PA was self-reported, which correlates only moderately with device-based measures and is susceptible to overestimation, potentially introducing non-differential misclassification that may bias associations toward the null [41, 42]. Although the HUNT PA questionnaires have demonstrated acceptable validity in Norwegian adult populations [43, 44], they have not been specifically validated in older adults. However, PA assessments at HUNT1, HUNT2, and HUNT3 were conducted when participants were in mid- to late adulthood rather than in advanced age, which may reduce the impact of age-related recall bias. Second, our assessment of diet quality was limited and varied across HUNT waves, relying on crude proxies (cooking fat type in HUNT2 and fruit/vegetable intake in HUNT3). This may not have adequately captured important dietary factors relevant to sarcopenia, particularly protein intake and overall dietary patterns. Third, the PA questionnaire used in HUNT2 differed from those used in HUNT1 and HUNT3, potentially introducing measurement inconsistency across time points. While all questionnaires assessed activity duration and intensity, differences in question format and response categories may have contributed to some misclassification. However, our categorisation based on meeting or not meeting recommended PA levels likely reduced the impact of such heterogeneity. At the same time, this binary approach enhances comparability with public health guidelines and clinical interpretability but inevitably reduces variability in activity volume, intensity, and type, which may have attenuated observed associations. Fourth, we used bioelectrical impedance to estimate body composition. Bioelectrical impedance analysis algorithms typically assume constant hydration status [45]. However, we did not confirm hydration status or ask participants to void their bladders before assessment, which may have affected measurement validity. Nevertheless, as highlighted by previous cohorts [46], this limitation likely has minimal influence on our findings and reflects real-world clinical practice scenarios where bioelectrical impedance analysis is commonly used due to its accessibility and practicality. Fifth, a substantial proportion of HUNT4 70+ participants were excluded due to missing PA or sarcopenia data (*n* = 2919). Excluded participants were older (mean age 79.8 vs. 76.4 years), more likely to be female (61.4% vs. 52.5%), and had substantially lower grip strength (28.0 vs. 34.0 kg), slower sit-to-stand performance (13.4 vs. 11.4 s), and reduced gait speed (0.85 vs. 1.01 m/s), indicating that the analytical sample underrepresents the most frail and least active individuals. This selection and survivor bias likely attenuates the observed associations, suggesting that our findings may represent conservative estimates of the true relationship between long-term PA patterns and sarcopenia risk. Similarly, individuals who were most persistently inactive may have experienced higher premature mortality before HUNT4, further contributing to survival bias. Sixth, the relatively low participation rate in the most active categories, particularly those active at all three time points, limited statistical power for some analyses. Seventh, residual confounding cannot be entirely ruled out despite adjustments for lifestyle and health factors. Reverse causation (i.e., sarcopenia leading to physical inactivity) is also possible given the observational study design. Finally, our findings may have limited generalizability to populations with different ethnic backgrounds, given that the HUNT study included a relatively ethnically homogenous population [47]. Genetic, cultural, and environmental factors may influence both PA patterns and sarcopenia risk, potentially affecting the magnitude of associations observed in other populations.

## Conclusion

This study suggests an inverse dose-response relationship between PA and sarcopenia, wherein each additional time point of adherence to the PA guidelines was associated with progressively lower odds of sarcopenia later in life. The association was more pronounced for confirmed versus probable sarcopenia. While PA patterns over time appeared relevant, the observational nature of the data precludes conclusions about whether being active at specific life stages matters more than others, and these findings should be interpreted with caution. Overall, our results support the potential importance of both initiating and maintaining sufficient PA from mid-life into older age to reduce the likelihood of developing sarcopenia after age 70.

## Supplementary Information

Supplementary Material 1.

## Data Availability

The data used in this study are from the HUNT database and are not publicly available due to privacy restrictions and data protection regulations. Researchers can access the data following approval of applications to the Regional Committees for Medical Health Research Ethics, HUNT.

## Abbreviations

BMI: Body Mass Index
COPD: Chronic Obstructive Pulmonary Disease
CVD: Cardiovascular Disease
EWGSOP: The European Working Group on Sarcopenia
HGS: Handgrip strength
HUNT: The Trøndelag Health Study
PA: Physical Activity
SMI: Skeletal Muscle Index
5-STS: Five times sit-to-stand
WHO: World Health Organization

## References

[1] Cruz-Jentoft AJ, Baeyens JP, Bauer JM, Boirie Y, Cederholm T, Landi F, et al. Sarcopenia: European consensus on definition and diagnosis: Report of the European Working Group on Sarcopenia in Older People. Age Ageing. 2010;39(4):412–23. 20392703 10.1093/ageing/afq034 PMC2886201

[2] Cruz-Jentoft AJ, Bahat G, Bauer J, Boirie Y, Bruyère O, Cederholm T, et al. Sarcopenia: revised European consensus on definition and diagnosis. Age Ageing. 2019;48(1):16–31. 30312372 10.1093/ageing/afy169 PMC6322506

[3] Kirk B, Zanker J, Bani Hassan E, Bird S, Brennan-Olsen S, Duque G. Sarcopenia Definitions and Outcomes Consortium (SDOC) Criteria are Strongly Associated With Malnutrition, Depression, Falls, and Fractures in High-Risk Older Persons. J Am Med Dir Assoc. 2021;22(4):741–5. 32771358 10.1016/j.jamda.2020.06.050

[4] Fielding RA, Vellas B, Evans WJ, Bhasin S, Morley JE, Newman AB, et al. Sarcopenia: an undiagnosed condition in older adults. Current consensus definition: prevalence, etiology, and consequences. International working group on sarcopenia. J Am Med Dir Assoc. 2011;12(4):249–56. 21527165 10.1016/j.jamda.2011.01.003 PMC3377163

[5] Wallengren O, Bosaeus I, Frändin K, Lissner L, Falk Erhag H, Wetterberg H, et al. Comparison of the 2010 and 2019 diagnostic criteria for sarcopenia by the European Working Group on Sarcopenia in Older People (EWGSOP) in two cohorts of Swedish older adults. BMC Geriatr. 2021;21(1):1–12. 34702174 10.1186/s12877-021-02533-y PMC8547086

[6] Ethgen O, Beaudart C, Buckinx F, Bruyère O, Reginster JY. The Future Prevalence of Sarcopenia in Europe: A Claim for Public Health Action. Calcif Tissue Int. 2017;100(3):229–34. 28012107 10.1007/s00223-016-0220-9 PMC5313588

[7] Bull FC, Al-Ansari SS, Biddle S, Borodulin K, Buman MP, Cardon G, et al. World Health Organization 2020 guidelines on physical activity and sedentary behaviour. Br J Sports Med. 2020;54(24):1451. 33239350 10.1136/bjsports-2020-102955 PMC7719906

[8] Strain T, Flaxman S, Guthold R, Semenova E, Cowan M, Riley LM, et al. National, regional, and global trends in insufficient physical activity among adults from 2000 to 2022: a pooled analysis of 507 population-based surveys with 5·7 million participants. Lancet Glob Health. 2024;12(8):e1232–43. 38942042 10.1016/S2214-109X(24)00150-5 PMC11254784

[9] Sánchez-Sánchez JL, He L, Morales JS, de Souto Barreto P, Jiménez-Pavón D, Carbonell-Baeza A, et al. Association of physical behaviours with sarcopenia in older adults: a systematic review and meta-analysis of observational studies. Lancet Healthy Longev. 2024;5(2):e108–19. 38310891 10.1016/S2666-7568(23)00241-6

[10] Aggio DA, Sartini C, Papacosta O, Lennon LT, Ash S, Whincup PH, et al. Cross-sectional associations of objectively measured physical activity and sedentary time with sarcopenia and sarcopenic obesity in older men. Prev Med. 2016;91:264–72. 27575317 10.1016/j.ypmed.2016.08.040 PMC5061552

[11] Steffl M, Bohannon RW, Sontakova L, Tufano JJ, Shiells K, Holmerova I. Relationship between sarcopenia and physical activity in older people: a systematic review and meta-analysis. Clin Interv Aging. 2017;12:835–45. 28553092 10.2147/CIA.S132940 PMC5441519

[12] Beckwée D, Delaere A, Aelbrecht S, Baert V, Beaudart C, Bruyere O, et al. Exercise Interventions for the Prevention and Treatment of Sarcopenia. A Systematic Umbrella Review. J Nutr Health Aging. 2019;23(6):494–502. 31233069 10.1007/s12603-019-1196-8 PMC12280365

[13] Fiatarone MA, Marks EC, Ryan ND, Meredith CN, Lipsitz LA, Evans WJ. High-Intensity Strength Training in Nonagenarians: Effects on Skeletal Muscle. JAMA. 1990;263(22):3029–34. 2342214

[14] Chen N, He X, Feng Y, Ainsworth BE, Liu Y. Effects of resistance training in healthy older people with sarcopenia: a systematic review and meta-analysis of randomized controlled trials. Eur Rev Aging Phys Act. 2021;18(1):23. 34763651 10.1186/s11556-021-00277-7 PMC8588688

[15] Shen Y, Shi Q, Nong K, Li S, Yue J, Huang J, et al. Exercise for sarcopenia in older people: A systematic review and network meta-analysis. J Cachexia Sarcopenia Muscle. 2023;14(3):1199–211. 37057640 10.1002/jcsm.13225 PMC10235889

[16] Dent E, Morley JE, Cruz-Jentoft AJ, Arai H, Kritchevsky SB, Guralnik J, et al. International Clinical Practice Guidelines for Sarcopenia (ICFSR): Screening, Diagnosis and Management. J Nutr Health Aging. 2018;22(10):1148–61. 30498820 10.1007/s12603-018-1139-9 PMC12280515

[17] QuickStats. Percentage* of Adults Aged ≥ 18 Years Who Met the Federal Guidelines for Muscle-Strengthening Physical Activity,† by Age Group and Sex - National Health Interview Survey, United States, 2020§. MMWR Morb Mortal Wkly Rep. 2022;71(18):642. 35511726 10.15585/mmwr.mm7118a6 PMC9098242

[18] Bennie JA, De Cocker K, Smith JJ, Wiesner GH. The epidemiology of muscle-strengthening exercise in Europe: A 28-country comparison including 280,605 adults. PLoS ONE. 2020;15(11):e0242220. 33237930 10.1371/journal.pone.0242220 PMC7688125

[19] Ren Z, Zhang Y, Drenowatz C, Eather N, Hong J, Wang L, et al. How many adults have sufficient muscle-strengthening exercise and the associated factors: A systematic review consisting of 2,629,508 participants. J Exerc Sci Fit. 2024;22(4):359–68. 39040428 10.1016/j.jesf.2024.06.002 PMC11261455

[20] Engberg E, Alen M, Kukkonen-Harjula K, Peltonen JE, Tikkanen HO, Pekkarinen H. Life Events and Change in Leisure Time Physical Activity. Sports Med. 2012;42(5):433–47. 22512413 10.2165/11597610-000000000-00000

[21] Confortin SC, Ono LM, Barbosa AR, d’Orsi E. Sarcopenia and its association with changes in socioeconomic, behavioral, and health factors: the EpiFloripa Elderly Study. Cad Saude Publica. 2018;34(12):12–24. 10.1590/0102-311X00164917 30517315

[22] Rantanen T, Era P, Heikkinen E. Physical Activity and the Changes in Maximal Isometric Strength in Men and Women from the Age of 75 to 80 Years. J Am Geriatr Soc. 1997;45(12):1439–45. 9400552 10.1111/j.1532-5415.1997.tb03193.x

[23] Yerrakalva D, Hajna S, Khaw KT, Griffin SJ, Brage S. Prospective associations between changes in physical activity and sedentary time and subsequent lean muscle mass in older English adults: the EPIC-Norfolk cohort study. Int J Behav Nutr Phys Act. 2024;21(1):1–12. 38279174 10.1186/s12966-023-01547-6 PMC10811887

[24] Hughes VA, Frontera WR, Roubenoff R, Evans WJ, Fiatarone Singh MA. Longitudinal changes in body composition in older men and women: Role of body weight change and physical activity. Am J Clin Nutr. 2002;76(2):473–81. 12145025 10.1093/ajcn/76.2.473

[25] Zhao X, Liu D, Zhang H, Shen S, Zhang N, Pan Y, et al. Associations of physical activity intensity, frequency, duration, and volume with the incidence of sarcopenia in middle-aged and older adults: a 4-year longitudinal study in China. BMC Geriatr. 2024;24(1):258. 38493082 10.1186/s12877-024-04873-x PMC10944603

[26] Mijnarends DM, Koster A, Schols JM, Meijers JM, Halfens RJ, Gudnason V, et al. Physical activity and incidence of sarcopenia: the population-based AGES-Reykjavik Study. Age Ageing. 2016;45(5):614–20. 27189729 10.1093/ageing/afw090 PMC5027639

[27] Skjellegrind HK, Thingstad P, Gjøra L, Kolberg M, Kjelvik G, Ernstsen L, et al. Cohort profile update: HUNT4 70+. Int J Epidemiol. 2026;55(2):dyag011. 10.1093/ije/dyag011 41709676

[28] Kirk B, Cawthon PM, Arai H, Ávila-Funes JA, Barazzoni R, Bhasin S, et al. The conceptual definition of sarcopenia: delphi consensus from the global leadership initiative in sarcopenia (GLIS). Age Ageing. 2024;53(3):afae052. 10.1093/ageing/afae052 PMC10960072 38520141

[29] Johansson J, Grimsgaard S, Strand BH, Sayer AA, Cooper R. Comparing associations of handgrip strength and chair stand performance with all-cause mortality—implications for defining probable sarcopenia: the Tromsø Study 2015–2020. BMC Med. 2023;21(1):451. 37981689 10.1186/s12916-023-03172-3 PMC10659040

[30] Berg J, Nauman J, Wisløff U. Normative values for body composition in 22,191 healthy Norwegian adults 20–99 years: The HUNT4 study. Prog Cardiovasc Dis. 2024;85:82–92. 38925258 10.1016/j.pcad.2024.06.002

[31] Yuan S, Larsson SC. Epidemiology of sarcopenia: Prevalence, risk factors, and consequences. Metabolism. 2023;144:155533. 36907247 10.1016/j.metabol.2023.155533

[32] Cooper A, Lamb M, Sharp SJ, Simmons RK, Griffin SJ. Bidirectional association between physical activity and muscular strength in older adults: Results from the UK Biobank study. Int J Epidemiol. 2017;46(1):141–8. 27209633 10.1093/ije/dyw054 PMC5407153

[33] Cruz-Jentoft AJ, Bahat G, Bauer J, Boirie Y, Bruyère O, Cederholm T, et al. Sarcopenia: revised European consensus on definition and diagnosis. Age Ageing. 2018;48(1):16–31. 10.1093/ageing/afy169 PMC6322506 30312372

[34] Wang K, Wang X, Wang Y. Factors, mechanisms and improvement methods of muscle strength loss. Front Cell Dev Biol. 2024;12:1509519. 39698495 10.3389/fcell.2024.1509519 PMC11653071

[35] Larsson L, Degens H, Li M, Salviati L, Lee YI, Thompson W, et al. Sarcopenia: Aging-Related Loss of Muscle Mass and Function. Physiol Rev. 2019;99(1):427–511. 30427277 10.1152/physrev.00061.2017 PMC6442923

[36] Mitchell WK, Williams J, Atherton P, Larvin M, Lund J, Narici M. Sarcopenia, dynapenia, and the impact of advancing age on human skeletal muscle size and strength; a quantitative review. Front Physiol. 2012;3:260. 22934016 10.3389/fphys.2012.00260 PMC3429036

[37] Goodpaster BH, Park SW, Harris TB, Kritchevsky SB, Nevitt M, Schwartz AV, et al. The Loss of Skeletal Muscle Strength, Mass, and Quality in Older Adults: The Health, Aging and Body Composition Study. Journals Gerontology: Ser A. 2006;61(10):1059–64. 10.1093/gerona/61.10.1059 17077199

[38] Voulgaridou G, Tyrovolas S, Detopoulou P, Tsoumana D, Drakaki M, Apostolou T et al. Diagnostic Criteria and Measurement Techniques of Sarcopenia: A Critical Evaluation of the Up-to-Date Evidence. Nutrients. 2024;16(3). 10.3390/nu16030436 PMC10856900 38337720

[39] Evans WJ. Skeletal muscle loss: cachexia, sarcopenia, and inactivity. Am J Clin Nutr. 2010;91(4):s1123–7. 10.3945/ajcn.2010.28608A 20164314

[40] Bunout D, Barrera G, Hirsch S, Jimenez T, de la Maza MP. Association between activity energy expenditure and peak oxygen consumption with sarcopenia. BMC Geriatr. 2018;18(1):298. 30509203 10.1186/s12877-018-0993-y PMC6276239

[41] Lee PH, Macfarlane DJ, Lam TH, Stewart SM. Validity of the international physical activity questionnaire short form (IPAQ-SF): A systematic review. Int J Behav Nutr Phys Activity. 2011;8(1):115. 10.1186/1479-5868-8-115 PMC3214824 22018588

[42] Dyrstad SM, Hansen BH, Holme IM, Anderssen SA. Comparison of self-reported versus accelerometer-measured physical activity. Med Sci Sports Exerc. 2014;46(1):99–106. 23793232 10.1249/MSS.0b013e3182a0595f

[43] Kurtze N, Rangul V, Hustvedt BE, Flanders WD. Reliability and validity of self-reported physical activity in the Nord-Trøndelag Health Study: HUNT 1. Scand J Public Health. 2008;36(1):52–61. 18426785 10.1177/1403494807085373

[44] Kurtze N, Rangul V, Hustvedt BE, Flanders WD. Reliability and validity of self-reported physical activity in the Nord-Trøndelag Health Study (HUNT 2). Eur J Epidemiol. 2007;22(6):379–87. 17356925 10.1007/s10654-007-9110-9

[45] Kyle UG, Bosaeus I, De Lorenzo AD, Deurenberg P, Elia M, Gómez JM, et al. Bioelectrical impedance analysis—part I: review of principles and methods. Clin Nutr. 2004;23(5):1226–43. 15380917 10.1016/j.clnu.2004.06.004

[46] Lee M-M, Jebb SA, Oke J, Piernas C. Reference values for skeletal muscle mass and fat mass measured by bioelectrical impedance in 390 565 UK adults. J Cachexia Sarcopenia Muscle. 2020;11(2):487–96. 31943835 10.1002/jcsm.12523 PMC7113534

[47] Åsvold BO, Langhammer A, Rehn TA, Kjelvik G, Grøntvedt TV, Sørgjerd EP, et al. Cohort Profile Update: The HUNT Study, Norway. Int J Epidemiol. 2023;52(1):e80–91. 35578897 10.1093/ije/dyac095 PMC9908054

