# Supplementary material for: Physical activity patterns from mid- to late adulthood and risk of sarcopenia in older adults: the HUNT study
Source: BMC Geriatr. 2026 Jun 4;26:1027. doi: 10.1186/s12877-026-07747-6 (PMC13459403; doi:10.1186/s12877-026-07747-6)
Supplement: Supplementary file 1 [file 12877_2026_7747_MOESM1_ESM.docx]

### Additional file 1: Participant characteristics at HUNT4 by sarcopenia group and sex, across all HUNT waves.

|  | **Men (n = 3361)** | | | **Women (n = 4260)** | | |
| --- | --- | --- | --- | --- | --- | --- |
| **Variable** | **No sarcopenia  (n = 1781)** | **Probable sarcopenia  (n = 1301)** | **Confirmed sarcopenia  (n = 279)** | **No sarcopenia  (n = 2335)** | **Probable sarcopenia  (n = 1755)** | **Confirmed sarcopenia  (n = 170)** |
| **Age (years)** | 75.2 (4.2) | 79.3 (6.3) | 82.2 (6.0) | 75.7 (4.6) | 80.9 (6.8) | 80.0 (6.3) |
| **Years of completed education, n (%)**  **7-10 years**  **11-13 years**  **> 13 years**  *Missing, n (%)* | 326 (18.5)  867 (49.2)  569 (32.3)  *19 (1.1)* | 307 (26.1)  607 (51.6)  263 (22.3)  *124 (9.5)* | 76 (27.3)  139 (50.0)  63 (22.7)  *1 (0.4)* | 705 (30.8)  1016 (44.4)  567 (24.8)  *47 (2.0)* | 705 (47.1)  594 (39.7)  197 (13.2)  *259 (14.8)* | 74 (44.3)  64 (38.3)  29 (17.4)  *3 (1.8)* |
| **Current or previous daily smoker, n (%)**  *Missing, n (%)* | 1049 (59.4)  *14 (0.8)* | 767 (65.1)  *122 (9.4)* | 178 (64.0)  *1 (0.4)* | 1164 (50.9)  *47 (2.0)* | 696 (46.0)  *243 (13.8)* | 91 (54.2)  *2 (1.2)* |
| **Weekly alcohol consumption, n (%)**  **Low: < 2 units**  **Moderate: 2-3 units**  **High: ≥ 4 units**  *Missing, n (%)* | 549 (33.8)  551 (33.9)  524 (32.3)  *157 (8.8)* | 462 (46.6)  296 (29.9)  223 (23.5)  *310 (23.8)* | 120 (53.3)  63 (28.0)  42 (18.7)  *54 (19.4)* | 1005 (53.5)  549 (29.2)  323 (17.2)  *458 (19.6)* | 824 (73.4)  211 (18.8)  87 (7.8)  *633 (36.1)* | 86 (64.7)  37 (27.8)  10 (7.5)  *37 (21.8)* |
| **Hypertension, n (%)**  *Missing, n (%)* | 1278 (82.5)  *232 (13.0)* | 906 (83.6)  *217 (16.7)* | 188 (79.7)  *43 (15.4)* | 1682 (83.2)  *314 (13.4)* | 1223 (85.3)  *322 (18.3)* | 128 (82.6)  *15 (8.8)* |
| **Diabetes (HUNT3), n (%)** | 98 (5.5) | 130 (10.0) | 20 (7.2) | 108 (4.6) | 154 (8.8) | 7 (4.1) |
| **Body mass index (kg/m^2^)**  *Missing, n (%)* | 27.2 (3.4)  *27 (1.5)* | 28.0 (4.0)  *86 (6.6)* | 24.1 (2.6)  *0 (0.0)* | 26.8 (4.5)  *31 (1.3)* | 27.9 (5.0)  *145 (8.3)* | 22.3 (3.2)  *0 (0.0)* |
| **Body mass index (kg/m^2^) category, n (%)**  **Underweight: < 18.5**  **Normal weight: 18.5-24.9**  **Overweight: 25-29.9**  **Obese: ≥ 30**  *Missing, n (%)* | 2 (0.1)  472 (26.9) 950 (54.2)  330 (18.8)  *27 (1.5)* | 3 (0.2)  258 (21.2) 622 (51.2)  332 (27.3)  *86 (6.6)* | 3 (1.1)  171 (61.3)  103 (36.9)  2 (0.7)  *0 (0.0)* | 28 (1.2)  815 (35.4) 941 (40.8)  520 (22.6)  *31 (1.3)* | 21 (1.3)  435 (27.0) 690 (42.9)  464 (28.8)  *145 (8.3)* | 19 (11.2)  120 (70.6)  29 (17.1)  2 (1.2)  *0 (0.0)* |
| **Handgrip strength (kg)**  *Missing, n (%)* | 46.5 (6.1)  *119 (6.7)* | 35.3 (8.7)  *43 (3.3)* | 33.1 (6.1)  *10 (3.6)* | 28.2 (4.0)  *174 (7.5)* | 20.6 (6.0)  *91 (5.2)* | 20.1 (4.2)  *6 (3.5)* |
| **Five times sit-to-stand (seconds)**  ***Not able to perform test, n (%)***  *Missing, n (%)* | 9.6 (1.9)  0 (0.0)  *81 (4.5)* | 13.4 (3.9)  309 (23.8)  *13 (1.0)* | 13.2 (4.4)  42 (15.1)  *2 (0.7)* | 10.6 (2.0)  0 (0.0)  *121 (5.2)* | 16.0 (6.3)  609 (34.7)  *14 (0.8)* | 14.0 (4.2)  22 (12.9)  *0 (0.0)* |
| **Skeletal muscle index (kg/m^2^)**  *Missing, n (%)* | 10.81 (0.89)  *133 (7.5)* | 10.69 (0.80)  *432 (33.2)* | 9.00 (0.48)  *0 (0.0)* | 8.90 (0.85)  *260 (11.1)* | 8.86 (0.79)  *821 (46.8)* | 7.23 (0.36)  *0 (0.0)* |
| **Gait speed (m/s)**  ***Not able to perform test, n (%)***  *Missing, n (%)* | 1.08 (0.24)  0 (0.0)  *118 (6.6)* | 0.87 (0.26)  97 (7.5)  *59 (4.5)* | 0.89 (0.23)  4 (1.4)  *10 (3.6)* | 1.06 (0.24)  2 (0.1)  *168 (7.2)* | 0.76 (0.27)  172 (9.8)  *84 (4.8)* | 0.86 (0.26)  4 (2.4)  *2 (1.2)* |

*Data are presented as mean (SD) unless otherwise stated.*

### Additional file 2: Participant characteristics at HUNT4 in included versus excluded participants.

| **Variable** | **Included (n = 4702)** | **Excluded (n = 2919)** |
| --- | --- | --- |
| **Age (years)**  *Missing, n (%)* | 76.4 (5.0)  *13 (0.3)* | 79.8 (7.0)  *336 (11.5)* |
| **Female, n (%)** | 2468 (52.5) | 1792 (61.4) |
| **Years of completed education, n (%)**  **7-10 years**  **11-13 years**  **> 13 years**  *Missing, n (%)* | 1236 (26.5)  2182 (46.8)  1248 (26.7)  *36 (0.8)* | 957 (38.2)  1105 (44.2)  440 (17.6)  *417 (14.3)* |
| **Current or previous daily smoker, n (%)**  *Missing, n (%)* | 2540 (54.6)  *47 (1.0)* | 1405 (55.4)  *382 (13.1)* |
| **Weekly alcohol consumption, n (%)**  **Low: < 2 units**  **Moderate: 2-3 units**  **High: ≥ 4 units**  *Missing, n (%)* | 1900 (47.0)  1240 (30.7)  899 (22.3)  *663 (14.1)* | 1146 (59.3)  467 (24.2)  320 (16.6)  *986 (33.8)* |
| **Hypertension, n (%)**  *Missing, n (%)* | 3414 (82.4)  *559 (11.9)* | 1991 (85.3)  *584 (20.0)* |
| **Diabetes (HUNT3), n (%)**  *Missing, n (%)* | 293 (6.2)  *0 (0.0)* | 224 (7.7)  *2 (0.1)* |
| **Body mass index (kg/m^2^)**  *Missing, n (%)* | 27.1 (4.1)  *0 (0.0)* | 27.2 (4.8)  *289 (9.9)* |
| **Body mass index category, n (%)**  **Underweight: < 18.5 kg/m^2^**  **Normal weight: ≤ 24.9 kg/m^2^**  **Overweight: 25-29.9 kg/m^2^**  **Obese: ≥ 30 kg/m^2^**  *Missing, n (%)* | 38 (0.8)  1437 (30.6)  2213 (47.1)  1650 (21.6)  *0 (0.0)* | 38 (1.4)  834 (31.7)  1122 (42.7)  636 (24.2)  *289 (9.9)* |
| **Handgrip strength (kg)**  ***Not able to perform test, n (%)*** *Missing, n (%)* | 34.0 (10.7)  0 (0.0) *0 (0.0)* | 28.0 (11.0)  144 (4.9) *443 (15.2)* |
| **Five times sit-to-stand (seconds)**  ***Not able to perform test, n (%)***  *Missing, n (%)* | 11.4 (3.5)  220 (4.7)  *0 (0.0)* | 13.4 (6.2)  762 (26.1)  *231 (7.9)* |
| **Skeletal muscle index (kg/m^2^)**  *Missing, n (%)* | 9.66 (1.30)  *0 (0.0)* | 9.57 (1.31)  *1646 (56.4)* |
| **Gait speed (m/s)**  ***Not able to perform test, n (%)***  *Missing, n (%)* | 1.01 (0.25)  29 (0.6)  *0 (0.0)* | 0.85 (0.31)  250 (8.6)  *309 (10.6)* |

*Data are presented as mean (SD) unless otherwise stated.*

### Additional file 3: Physical activity patterns and the odds of sarcopenia in self-reported healthy participants.

| PHYSICAL ACTIVITY PATTERNS  HUNT1, HUNT2, HUNT3 | **ODDS OF SARCOPENIA AT HUNT4** | | | |
| --- | --- | --- | --- | --- |
|  | **Probable sarcopenia  normative values** | | **Confirmed sarcopenia  normative values** | |
|  | OR (95% CI)  Model 1 | OR (95% CI)  Model 2 | OR (95% CI)  Model 1 | OR (95% CI)  Model 2 |
| **Always inactive**  (n = 1659) | 1.00 (Reference) | 1.00 (Reference) | 1.00 (Reference) | 1.00 (Reference) |
| **Becoming inactive** ^a^  (n = 80) | 0.83 (0.50 - 1.38) | 0.88 (0.53 - 1.48) | 1.18 (0.49 - 2.83) | 1.17 (0.46 - 2.97) |
| **Fluctuating** ^b^  (n = 478) | 0.78 (0.62 - 0.99) | 0.86 (0.68 - 1.10) | 0.90 (0.57 - 1.41) | 0.76 (0.47 - 1.22) |
| **Becoming active** ^c^  (n = 454) | 0.69 (0.54 - 0.87) | 0.74 (0.58 - 0.95) | 0.83 (0.52 - 1.32) | 0.67 (0.41 - 1.08) |
| **Always active**  (n = 75) | 0.81 (0.48 - 1.36) | 0.92 (0.54 - 1.57) | 0.35 (0.10 - 1.21) | 0.26 (0.07 - 0.91) |

*^a^ = active at HUNT1 but inactive by HUNT3 (either active or inactive at HUNT2), ^b^ = a non-sequential pattern: active-inactive-active or inactive-active-inactive, ^c^ = inactive at HUNT1 but active by HUNT3 (either active or inactive at HUNT2).
Model 1: adjusted for sex (male, female) and age. Model 2: further adjusted for BMI category, education category, self-reported health status (history of angina pectoris, myocardial infarction, heart failure, atrial fibrillation, stroke, or COPD), smoking (yes, no), hypertension (yes, no), diabetes (yes, no), weekly alcohol consumption, and diet (healthy, poor). CI: confidence interval.*

### Additional file 4: Physical activity patterns and the odds of sarcopenia, excluding participants with mobility impairments at HUNT3.

| PHYSICAL ACTIVITY PATTERNS  HUNT1, HUNT2, HUNT3 | **ODDS OF SARCOPENIA AT HUNT4** | | | |
| --- | --- | --- | --- | --- |
|  | **Probable sarcopenia  normative values** | | **Confirmed sarcopenia  normative values** | |
|  | OR (95% CI)  Model 1 | OR (95% CI)  Model 2 | OR (95% CI)  Model 1 | OR (95% CI)  Model 2 |
| **Always inactive**  (n = 2178) | 1.00 (Reference) | 1.00 (Reference) | 1.00 (Reference) | 1.00 (Reference) |
| **Becoming inactive** ^a^  (n = 110) | 0.70 (0.45 - 1.09) | 0.77 (0.49 - 1.20) | 0.82 (0.36 - 1.84) | 0.79 (0.34 - 1.83) |
| **Fluctuating** ^b^  (n = 645) | 0.78 (0.64 - 0.96) | 0.87 (0.71 - 1.06) | 1.05 (0.74 - 1.48) | 0.89 (0.62 - 1.28) |
| **Becoming active** ^c^  (n = 584) | 0.69 (0.56 - 0.85) | 0.77 (0.62 - 0.95) | 0.77 (0.52 - 1.14) | 0.65 (0.43 - 0.97) |
| **Always active**  (n = 94) | 0.67 (0.41 - 1.07) | 0.82 (0.50 - 1.33) | 0.33 (0.10 - 1.09) | 0.20 (0.06 - 0.69) |

^a^ = inactive at HUNT1, active at HUNT2, inactive at HUNT3, or active at HUNT1, inactive at HUNT2, active at HUNT3, ^b^ = active at HUNT1, inactive at HUNT3 (either active or inactive at HUNT2), ^c^ = inactive at HUNT1, active at HUNT3 (either active or inactive at HUNT2).
Model 1: adjusted for sex (male, female) and age. Model 2: further adjusted for BMI category, education category, self-reported health status (history of angina pectoris, myocardial infarction, heart failure, atrial fibrillation, stroke, or COPD), smoking (yes, no), hypertension (yes, no), diabetes (yes, no), weekly alcohol consumption, and diet (healthy, poor). CI: confidence interval.

### Additional file 5: Physical activity patterns from HUNT1 to HUNT3 and the odds of sarcopenia.

| PHYSICAL ACTIVITY PATTERNS  HUNT1-HUNT3 | **ODDS OF SARCOPENIA AT HUNT4** | | | |
| --- | --- | --- | --- | --- |
|  | **Probable sarcopenia  normative values** | | **Confirmed sarcopenia  normative values** | |
|  | OR (95% CI)  Model 1 | OR (95% CI)  Model 2 | OR (95% CI)  Model 1 | OR (95% CI)  Model 2 |
| **Inactive, inactive**  (n = 3421) | 1.00 (Reference) | 1.00 (Reference) | 1.00 (Reference) | 1.00 (Reference) |
| **Active, inactive**  (n = 143) | 0.79 (0.55 - 1.16) | 0.83 (0.57 - 1.21) | 0.72 (0.36 - 1.45) | 0.73 (0.35 - 1.52) |
| **Inactive, active**  (n = 686) | 0.71 (0.59 - 0.85) | 0.78 (0.65 - 0.95) | 0.69 (0.48 - 0.98) | 0.59 (0.41 - 0.86) |
| **Always active**  (n = 135) | 0.83 (0.56 - 1.21) | 0.99 (0.67 - 1.45) | 0.41 (0.17 - 0.96) | 0.30 (0.12 - 0.74) |

Model 1: adjusted for sex (male, female) and age. Model 2: further adjusted for BMI category, education category, self-reported health status (history of angina pectoris, myocardial infarction, heart failure, atrial fibrillation, stroke, or COPD), smoking (yes, no), hypertension (yes, no), diabetes (yes, no), weekly alcohol consumption, and diet (healthy, poor). CI: confidence interval.

Additional file 6: Physical activity patterns and sarcopenia odds with 10 imputed datasets on all potential confounders.

| PHYSICAL ACTIVITY PATTERNS  HUNT1, HUNT2, HUNT3 | **ODDS OF SARCOPENIA AT HUNT4** | | | |
| --- | --- | --- | --- | --- |
|  | **Probable sarcopenia  normative values** | | **Confirmed sarcopenia  normative values** | |
|  | OR (95% CI)  Model 1 | OR (95% CI)  Model 2 | OR (95% CI)  Model 1 | OR (95% CI)  Model 2 |
| **Always inactive**  (n = 2914) | 1.00 (Reference) | 1.00 (Reference) | 1.00 (Reference) | 1.00 (Reference) |
| **Becoming inactive** ^a^  (n = 153) | 0.76 (0.53 - 1.09) | 0.80 (0.56 - 1.16) | 0.76 (0.40 - 1.45) | 0.76 (0.39 - 1.51) |
| **Fluctuating** ^b^  (n = 800) | 0.75 (0.63 - 0.90) | 0.83 (0.69 - 0.99) | 0.88 (0.65 - 1.20) | 0.81 (0.58 - 1.12) |
| **Becoming active** ^c^  (n = 722) | 0.65 (0.54 - 0.78) | 0.73 (0.61 - 0.88) | 0.65 (0.46 - 0.93) | 0.55 (0.38 - 0.80) |
| **Always active**  (n = 113) | 0.61 (0.39 - 0.94) | 0.75 (0.48 - 1.16) | 0.31 (0.11 - 0.89) | 0.22 (0.08 - 0.65) |

^a^ = active at HUNT1, inactive at HUNT3 (either active or inactive at HUNT2), ^b^ = inactive at HUNT1, active at HUNT2, inactive at HUNT3, or active at HUNT1, inactive at HUNT2, active at HUNT3, ^c^ = inactive at HUNT1, active at HUNT3 (either active or inactive at HUNT2).
Model 1: adjusted for sex (male, female) and age. Model 2: further adjusted for BMI category, education category, self-reported health status (history of angina pectoris, myocardial infarction, heart failure, atrial fibrillation, stroke, or COPD), smoking (yes, no), hypertension (yes, no), diabetes (yes, no), weekly alcohol consumption, and diet (healthy, poor). CI: confidence interval.

**Additional file 7: Physical activity patterns and odds of sarcopenia, including participants unable to perform grip strength test.**

| PHYSICAL ACTIVITY PATTERNS  HUNT1, HUNT2, HUNT3 | **ODDS OF SARCOPENIA AT HUNT4** | | | |
| --- | --- | --- | --- | --- |
|  | **Probable sarcopenia  normative values** | | **Confirmed sarcopenia  normative values** | |
|  | OR (95% CI)  Model 1 | OR (95% CI)  Model 2 | OR (95% CI)  Model 1 | OR (95% CI)  Model 2 |
| **Always inactive**  (n = 2705) | 1.00 (Reference) | 1.00 (Reference) | 1.00 (Reference) | 1.00 (Reference) |
| **Becoming inactive** ^a^  (n = 144) | 0.73 (0.50 - 1.07) | 0.78 (0.53 - 1.14) | 0.71 (0.35 - 1.44) | 0.70 (0.33 - 1.47) |
| **Fluctuating** ^b^  (n = 761) | 0.76 (0.64 - 0.91) | 0.84 (0.70 - 1.01) | 0.94 (0.68 - 1.29) | 0.84 (0.60 - 1.18) |
| **Becoming active** ^c^  (n = 688) | 0.66 (0.54 - 0.80) | 0.74 (0.61 - 0.90) | 0.68 (0.47 - 0.98) | 0.57 (0.39 - 0.83) |
| **Always active**  (n = 108) | 0.63 (0.41 - 0.98) | 0.78 (0.50 - 1.23) | 0.33 (0.12 - 0.94) | 0.22 (0.08 - 0.66) |

^a^ = active at HUNT1 but inactive by HUNT3 (either active or inactive at HUNT2), ^b^ = a non-sequential pattern: active-inactive-active or inactive-active-inactive, ^c^ = inactive at HUNT1 but active by HUNT3 (either active or inactive at HUNT2). Model 1: adjusted for sex (male, female) and age. Model 2: further adjusted for BMI category, education category, self-reported health status (history of angina pectoris, myocardial infarction, heart failure, atrial fibrillation, stroke, or COPD), smoking (yes, no), hypertension (yes, no), diabetes (yes, no), weekly alcohol consumption, and diet (healthy, poor). CI: confidence interval.
